# Supplementary material for: The causal relationship between the human gut microbiota and pyogenic arthritis: a Mendelian randomization study
Source: Front Cell Infect Microbiol. 2024 Nov 26;14:1452480. doi: 10.3389/fcimb.2024.1452480 (PMC11629706; doi:10.3389/fcimb.2024.1452480)
Supplement: Supplementary file 1 [file DataSheet1.zip › mendelian test/ebi-a-GCST90027474.csv_forest.pdf]

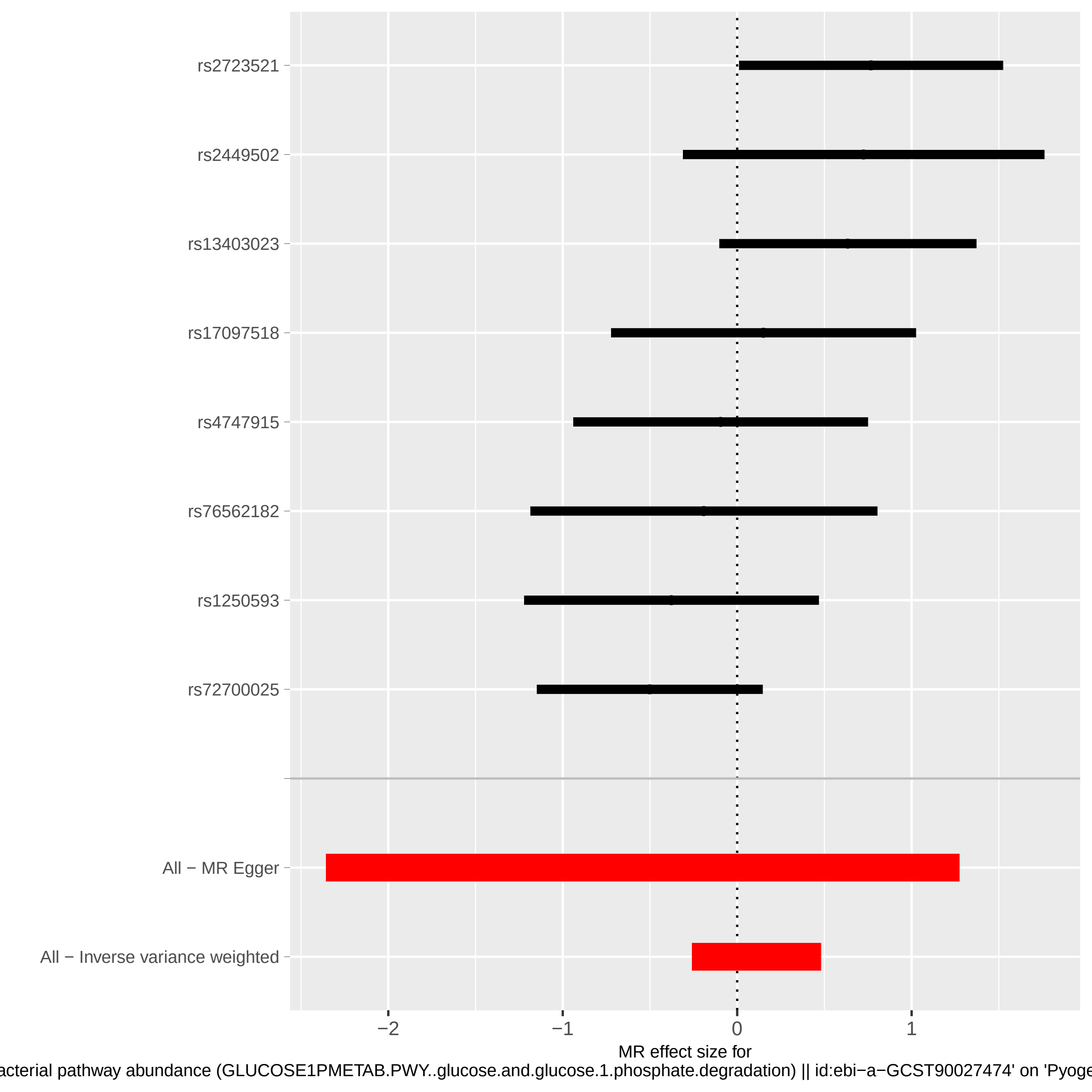

rs2723521
rs2449502
rs13403023
rs17097518
rs4747915
rs76562182
rs1250593
rs72700025
All − MR Egger
All − Inverse variance weighted
−2
−1
0
1
MR effect size for
acterial pathway abundance (GLUCOSE1PMETAB.PWY..glucose.and.glucose.1.phosphate.degradation) || id:ebi−a−GCST90027474' on 'Pyoge
